# 2D- and 3D-Based Intestinal Stem Cell Cultures for Personalized Medicine

**DOI:** 10.3390/cells7120225

**Published:** 2018-11-22

**Authors:** Yuan Liu, Ye-Guang Chen

**Affiliations:** The State Key Laboratory of Membrane Biology, Tsinghua-Peking Center for Life Sciences, School of Life Sciences, Tsinghua University, Beijing 100084, China; liuyuan14@mails.tsinghua.edu.cn

**Keywords:** intestinal stem cells, organoids, monolayer, cell culture, personalized medicine

## Abstract

Colorectal cancer (CRC) is one of the most common cancers that have high occurrence and death in both males and females. As various factors have been found to contribute to CRC development, personalized therapies are critical for efficient treatment. To achieve this purpose, the establishment of patient-derived tumor models is critical for diagnosis and drug test. The establishment of three-dimensional (3D) organoid cultures and two-dimensional (2D) monolayer cultures of patient-derived epithelial tissues is a breakthrough for expanding living materials for later use. This review provides an overview of the different types of 2D- and 3D-based intestinal stem cell cultures, their potential benefits, and the drawbacks in personalized medicine in treatment of the intestinal disorders.

## 1. Introduction

The intestine is a key organ that is responsible for food digestion, nutrients absorption, and waste excretion [1,2,3], with the capacity to sustain microbial and defense pathogens, as well as to regenerate following damage by inflammation or irradiation [4]. The intestinal epithelium, which is composed of a single layer of cells and forms the crypt–villus structure, is rapidly renewed, with a complete turnover every 4–5 days [3]. The self-renewal is driven by intestinal stem cells (ISCs), which are marked by Leucine-rich-repeat-containing G-protein-coupled receptor 5 (Lgr5) and localized at the base of the crypts and intermingled with Paneth cells that provide signals to support stem cell self-renewal and maintenance [5,6,7,8,9]. Along the villus, the epithelium consists of terminally differentiated functional cells, including enterocytes, enteroendocrine cells, goblet cells, tuft cells, and other cell types. These ISCs-derived cells move up toward the tip of villus during differentiation, and eventually die at the tip of villus and are shed off into the intestinal lumen [10,11]. Although there are no villus structures in the large intestine, the architecture of the epithelium is similar to the one of the small intestine, with the ISCs at the bottom of the crypts [3]. The self-renewal and differentiation of the ISCs are tightly controlled by the coordination of multiple niche factors [2,12], such as epidermal growth factor (EGF), Wnt ligands [13,14], bone morphogenetic proteins (BMPs) [15,16,17], Notch [18,19], and others [20], as well as Hippo/YAP signaling [21].

Colorectal cancer (CRC) progresses sequentially from adenomatous polyps to advanced adenomas, carcinomas, and adenocarcinomas, and gene mutations in multiple pathways have been reported in different stages of CRC development [20,22,23]. For instance, gain-of-function mutations have been observed in the various components of the Wnt/β-catenin pathway, such as *APC*, *CTNNB1*, *RNF43* and, while loss-of-function mutations are found to be associated with transforming growth factor β (TGF-β)/bone morphogenetic protein (BMP) signaling components such as *SMAD2*, *SMAD4*, *TGFBR2*, *BMPR2*, and *BMPR1A.* Gene mutations have also been reported in *KRAS*, *BRAF, TP53*, and DNA repair machinery. Different mutations cast complexity upon CRC treatment. In addition, ulcerative colitis (UC) and Crohn’s disease have abnormal immune responses in the colon and rectum, but whether these immune responses are a cause or a result of the disease is still under debate [24,25,26]. Although dextran sodium sulfate (DSS)-induced intestinal colitis in mice is a well-established and characterized model for acute intestinal injury and inflammation, this model, as well as the immune cells and epithelium co-culture system, have not yielded to effective therapies to treat inflammatory bowel colitis [27,28]. Furthermore, there are no specific treatments for enteric pathogen-caused illness [29,30].

Although chemotherapy and irradiation therapies are developing rapidly, gastroenterological disorders are still a major public health problem in the world due to the disease complexity. At the moment, most patients receive similar one-size-fits-all treatments. However, certain therapies work well for some patients, but they do not display promising results in others [31,32]. Therefore, personalized medicine or individualized treatments are in urgent demand to revolutionize future healthcare. To achieve this, precise patient-derived disease models are essential. Several patient-derived preclinical models have been established for intestinal disorders, including patient-derived tumor xenografts (PDX), culture systems of cancer cell lines, spheroids, and organoids and monolayers of intestinal epithelium and tumor tissues. These models have proven to be extremely valuable, not only for drug discovery and clinical trials, but also for a basic understanding of the fundamental mechanisms underlying tumorigenesis, inflammation, and interactions between the host–pathogen. This review summarizes the current 2D- or 3D-based intestinal epithelium culture systems, and discusses the advantages, caveats, potentials, and challenges, highlighting their applications in personalized medicine.

## 2. 3D-Based Organoid Culture System

Cell-based assays have been widely adopted in drug discovery for past decades. Historically, colon cancer cell lines, for instance HCT-116, HT-29 and SW480, have been used as the only practical option to discover drug candidates [31,32]. However, it has been demonstrated that the traditional culture system does not have sufficient clinical predictive power, due to the loss of tissue-specific architecture, the existence of gene mutations, and the lack of cell diversity in the intestine, therefore poorly recapturing tumor or inflammation conditions in patients [33]. Moreover, the necessity of adding three dimensions to the cell culture system that more closely resembles the natural environment, and its therapeutic potential, is being increasingly recognized for the future.

Scaffold-free and scaffold-based cultures are two major 3D culture systems. The scaffold-free system, like the sphere culture of the tumor cell lines in a low-attachment culture dish, suffers from similar defects as mentioned above [34]. Using droplets of a hanging drop plate, neural stem cells [35], mammary stem cells [36], corneal stem cells [37], and the embryoid bodies (EB) cultured from embryonic stem cells (ES) [38] could be cultivated in a spheroid, while it cannot be applied to the intestine. The other scaffold-based system, PDX models generated by transplanting the patient’s tumor cells to an immune-deficient mouse, could recapture some aspects of niche effects, such as the interactions of tumor cells with the surrounding stromal cells and immune cells; however, the nonmetastatic colorectal cancers fail to establish engraftments in mice [39]. A significant breakthrough was achieved by the establishment of three-dimensional (3D) organoid cultures of intestinal epithelium [40]. This culture system can rebuild the morphological, functional, and certain microenvironmental features of the intestine in vitro. The 3D organoid culture can mimic the in vivo physiology of the intestinal epithelium and it can therefore be used as a new tool for drug discovery and drug testing (Table 1).

The development of the murine intestinal organoid culture system by Clevers’ group in 2009 opened up a new era for the investigation of organ regeneration and homeostasis [41]. The successful establishment of the organoid culture system was based on the identification of the leucine-rich repeat-containing G protein-coupled receptor 5 (Lgr5) as a feasible marker of intestinal stem cells [42]. The intestinal epithelial organoids, also named “mini-gut”, can be cultured from the crypt epithelium, or even from single Lgr5^+^ cells [40]. Briefly, the isolated crypt epithelium or intestinal stem cells are embedded in Matrigel, a collagen- and laminin-rich extracellular matrix extract, and allowed to self-organize into a gut-like epithelial structure. The culture medium was supplemented with three growth factors: the Wnt agonist R-spondin1, the bone morphogenetic protein (BMP) antagonist Noggin, and epidermal growth factor (EGF) [40]. The 3D organotypic structures are very similar to that of normal intestinal epithelium in vivo, comprising of multiple crypt-like domains containing basal Lgr5^+^ cells interspersed with Paneth cells, and proliferative transient amplifying (TA) cells, enterocytes, goblet cells, and enteroendocrine cells [3,41,43]. Importantly, these cultured organoids retain their self-renewal and differentiation abilities, and their phenotype, karyotype, and gene expression profile are similar to the gut epithelium in vivo.

**Figure 1:**
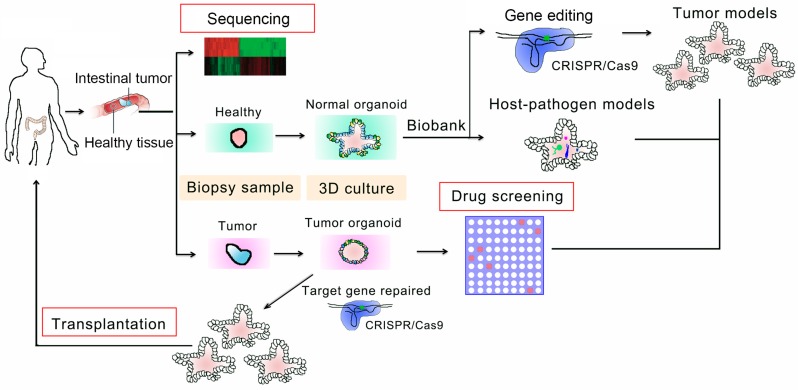
Applications of organoid technology for drug screening and therapy in personalized medicine. Gene mutations in patients’ tissues can be identified by genome or transcriptome sequencing. Meanwhile, the biopsy materials can be cultured in Matrigel to form organoids, which can be used to establish disease models via gene editing and co-culturing with niche cells and pathogens. The modified tumor organoids can be utilized for drug screening and testing. Gene mutations in organoids can be corrected by clustered regularly interspaced short palindromic repeats/CRISPR-associated 9 (CRISPR/Cas9)-mediated gene editing for transplantation.

After the 3D culture system for murine intestinal organoids was set up, more efforts have been made to adapt this system for human tissues, especially for patient-derived colon cancers [44] (Figure 1). In addition to the three growth factors R-spondin, Noggin, and EGF, the generation of patient-derived organoids requires other factors such as Wnt3a, gastrin, prostaglandin E_2_, nicotinamide, the TGF-β receptor inhibitor A83-01, and the p38 inhibitor SB202190 [43].

## 3. 2D-Based Culture System

Although 3D-based organoid culture technology has had a great impact on the in vitro study of gut epithelium biology and its therapeutic potential, the culture system faces significant challenges. Firstly, the self-renewing organoids embedded in Matrigel could mimic some of the in vivo epithelium features, but these organoids cultured as pure epithelium lack niche cells such as stromal cells, immune cells, and vasculature [45,46,47], and are unable to fully mimic the in vivo microenvironment. Secondly, some key cell types were not found in organoids; for example, secretin^+^ enteroendocrine cells were missing in the 3D culture [16,48]. Thirdly, the spheroidal structure of organoids restricts luminal access of exogenous compounds and pathogens, therefore restricting physiologically relevant studies on apical transporters, metabolic enzymes, and microbiota [49,50,51,52,53,54]. They could not be used for drug screening to treat UC or Crohn’s disease. Lastly, as the Matrigel is derived from animals with undefined hydrogel matrix compositions, it has limited broad applications for the 3D organoid culture system in mechanistic and clinic-related studies. To overcome some of these limitations, therefore, researchers have focused back on 2D monolayer culture systems.

Previous works had suggested that long-term culture of primary intestinal epithelium as a planar monolayer was impossible because of rapid stem cell loss and accelerated apoptosis. Recently, several groups have successfully established 2D monolayer culture systems of intestinal epithelia that can promote ISC maintenance [51,55,56,57,58,59,60].

**Figure 2:**
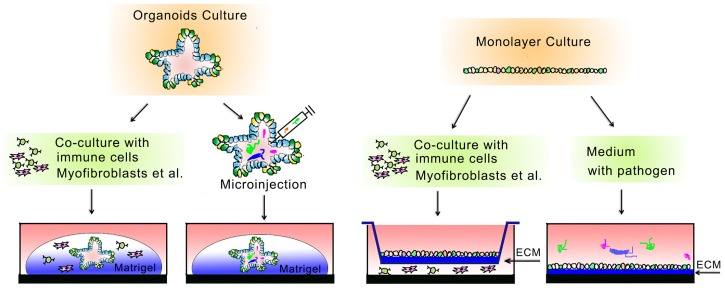
Models for intestinal epithelium-niche cells and epithelium–microbes/pathogen interactions. Both the 3D organoid culture and the transwell-based 2D monolayer culture can be used for the investigation of intestinal epithelium–niche cell interactions. To study the epithelium–microbe/pathogen interactions, microbes and pathogens can be introduced directly into the culture medium of 2D monolayer systems, while for the 3D cultures, pathogens need to be injected into the lumen of organoids to allow them to come into contact with the apical side of the epithelium.

Although different approaches have been used to culture intestinal epithelial monolayers (Table 1), a common method involves culturing cells on an extracellular matrix-coated surface. Use of a thin coating of type I collagen and recombinant human laminin is the most common choice, which has been shown to support the rapid expansion of proliferating monolayers of mice and human intestinal epithelium containing ISCs [57,60]. Although these approaches are appealing, the technical complexity associated with matrix neutralization of the acidic collagen solution with sodium hydroxide [60] or with intestinal subepithelial myofibroblast conditioned medium [57] may restrict the broad application of such systems. Based on the transwell system, several groups have described robust monolayer culture systems, which generate cells with the apical face of epithelium towards the culture medium [51,56,59]. Furthermore, a combination of the epithelial monolayer culture on the transwell insert and the niche cell culture on the transwell plate provides an effective tool to assess the effect of niche factors from immune cells or stromal cells in the intestine (Figure 2).

However, the Bolstering Lgr5 Transformational (BLT) sandwich culture system, consisting of a bottom collagen IV coating and a collagen I gel overlay, with intestinal epithelial cells between them [59], is still complex, and this may limit its application. Furthermore, mature secretory cell markers (Lyz1, Muc2, and Chga) were absent in the system established by Tong and colleagues. Similarly, the Paneth cell marker Lyz1 was not detected in the monolayer culture described by Puzan and colleagues [56]. A lack of important cell types raises the concern of whether these systems can recapture the pathophysiological features of the intestine in normal people and patients.

Most recently, we and Thorne et al. independently established simple and economical 2D monolayer culture systems that can recapture most of the features of 3D-cultured organoids and in vivo tissue [55,58]. The intestinal crypt epithelium is placed on a thin Matrigel layer on the culture dish. To maximize the expansion of Lgr5^+^ ISCs, the small molecules CHIR-99021 (inhibitor of GSK3 to activate Wnt/β-catenin signaling) and LDN-193189 (BMP type I receptor inhibitor) are added in the culture medium [55,58,59]. However, different from the system by Thorne et al., our system uses blebbistatin, which could improve the survival of cultured intestinal epithelium [61], and places Matrigel with uniform thickness, which could maintain the uniform growth of epithelial cells and reduce the cost of Matrigel.

Using a transwell-based 2D system, Wang and colleagues were able to establish a columnar epithelium with villus-like structures [62]. In their culture system, irradiated 3T3-J2 cells are seeded to transwell inserts coated with 20% Matrigel as a feeder layer. Then, human ISCs are placed onto the feeder layer in a medium containing R-spondin1, Jagged-1, Noggin, and other small molecules. More importantly, by removing the apical medium, the system allowed for columnar epithelium differentiation toward villus-like structures at the air–liquid interface. They further reported the cloning and propagation of ‘’ground state’’ human intestinal stem cells (ISC^GS^) that had higher clonogenicity compared with organoids.

A 2D-based monolayer culture allows for better imaging observation of the dynamics of Lgr5^+^ stem cells and the interplay between stem cells and differentiated cells. Moreover, in contrast to the inaccessibility of the apical domain of the epithelium in organoids, exposure of the surface in the monolayer provides a suitable system to investigate the pathogen–epithelial cell interaction [63]. However, one vital limitation of the all monolayer culture systems that needs to be solved is that it cannot be easily passaged and propagated [64]. Although efficient passaging between 2D and 3D can be achieved, 2D-to-2D passaging can be accomplished with a limited number of iterations, and long-term passaging needs further optimization [16,55,57,60].

## 4. Applications in Personalized Therapy

Development of stem-cell-derived culture technologies raises an unprecedented opportunity to reform the health care system by transforming disease modeling to the assessment of efficacy and safety profiles, including potential drug development, the cytotoxicity evaluation of new therapeutic compounds, personalized medicine, and transplantation-based therapies for end-stage diseases [44,65,66].

As 3D organoid culture is based on patient-specific tissues and can recapture in vivo-like complexity and architecture, it has been used to investigate the mechanisms of tissue homeostasis and disease development, including colorectal cancer and ulcerative colitis [67]. Many genetic gastroenterological disorders can be modeled with patient-derived or gene-edited organoids. For instance, Jarno et al. and Mami et al. have genetically edited the genome of normal human intestinal stem cells isolated from the patients by CRISPR/Cas9 technology to target the most highly mutated colorectal cancer hotspot (*APC*, *TP53*, *KRAS*, and *SMAD4*) [68,69]. Moreover, Verissimo and colleagues reported that patient-derived tumor organoids with a CRISPR/Cas9-introduced oncogenic KRAS mutation could be used to evaluate drug efficacy in a preclinical setting [70]. Therefore, these gene-edited organoids yield a new avenue by which to screen anticancer candidates in different stages of tumorigenesis.

Another important application of intestinal organoids is to investigate the pathogenesis of various infectious diseases, and to understand the dynamics of the host–microbe interaction [29,30]. By microinjection into the organoid lumen (Figure 2), the interaction of enteric pathogens with the human intestinal epithelia have already been demonstrated, such as *Vibrio cholera* [71], enteroaggregative *Escherichia coli* (*EAEC*), enteropathogenic *E. coli* (*EPEC*), enterohaemorrhagic *E. coli* (*EHEC*) [72], and rotavirus [73,74]. Similarly or even better, the 2D models can be used to explore the interaction between gut microbes and the intestinal epithelium, which could not only uncover the impact of microbes on epithelium homeostasis, but also help develop an effective and personalized treatment of the intestinal disorders.

In addition to the establishment of several specific disease models, organoids can be used for high-throughput drug screens [75]. A variety of organoid-based high-throughput screen systems have been established, such as organoid-on-chip [76,77]. As there is a strong correlation between gene mutation status and the therapeutic response, known as mutation-based drug sensitivity [78], the adoption of the tumor organoids has been used in precise cancer therapy. A good example was reported by van de Wetering and colleagues, who revealed that the organoids with *TP53* loss-of-function mutations displayed resistance to the MDM2 inhibitor Nutlin-3a using 20 colon tumor organoids [65]. Interestingly, an investigation of drug sensitivities of tumor-derived organoids against the library of 85 therapeutic compounds including chemotherapy and targeted therapy agents resulted in the identification of an effective treatment for each individual patient. Together, modeling specific and rare subtypes of cancer by the means of genetically engineered organoids could help to identify effective and personalized treatments [75,79].

Although much work has been done with 3D cultured organoids as disease models, drug screening, and personalized therapy, the 2D monolayer culture represents a transformative technology for personalized medicine applications that depend on drug and compound screening related to dietary and microbial metabolites. Parasites (*Cryptosporidium*) and microbes (*Shigella*, *E. coli*, *Clostridium difficile* and *Salmonella typhi*) can be introduced into the medium of 2D cultures directly. Using a 2D monolayer system, Wang et al. found that tannic acid could significantly inhibit intestinal epithelium growth in 2D monolayers, but not in 3D organoids, which may be due to exposure of the compound to different cell surfaces (apical side in 2D vs basolateral side in 3D) [60]. Moreover, the monolayer also provides a system for characterizing ion transport across the intestinal barrier [63]. Indeed, Kozuka et al. identified an inhibitor of potassium absorption in the murine distal colon using an epithelial monolayer culture [51]. Furthermore, the transwell-based monolayer culture is an adequate system for investigating the crosstalk between intestinal epithelial cells and niche cells (mesenchymal cells, immune cells, and myofibroblasts) as well as the enteric nervous system [56,80].

## 5. Challenges, Limitations, and Perspectives

As patient-derived organoids and monolayers are faithful replicas of the patient’s intestine epithelial tissues, these systems are great models that will undoubtedly facilitate diagnosis, molecule screening, drug testing, and transplantation as personalized approaches to treating intestine disorders. However, many challenges remain to meet the demands in quantity, quality, and processing robustness for commercialization and clinical trials.

For regeneration medicine, successful and efficient FDA-approved transplantation needs further improvement under culture conditions, including the animal-derived Matrigel and the high-cost of the growth factors. Several groups have used polyethylene glycol (PEG) and collagen to replace Matrigel as the supporting matrix in the culture [49,57,59,63], but the technical tediousness for handling PEG should not be neglected. Another big challenge is that niche reconstitution as the current culture system is designed for epithelial tissues. Considering the multi-functional and structurally complexity of the in vivo environment, niche cells including immune cells, stromal cells and other cell types, and vasculature, should be taken into account to better reflect the pathophysiological conditions. It is extremely important to understand the mechanism of inflammatory intestinal disorders, and ultimately to design efficient therapies to treat these untreatable diseases. For instance, the inclusion of niche cells would facilitate the screening of immunotherapy drugs and/or stromal-targeted agents. In this regard, the transwell-based 2D monolayer culture and microfluidics technology may provide some advantages of co-culture of epithelial cells and other types of cells to reconstitute the microenvironment [56,81]. Co-culture with niche cells has also been attempted in 3D systems [45], but the complex nature and high cost of 3D cultures limit their application, especially for a large-scale expansion. Recently, we have successfully established a growth factor-free culture system of murine intestinal organoids with two small molecules, which lights hope in this direction [82]. Furthermore, safe application in the clinic requires the long-term maintenance of the genome, without mutations or epigenetic changes.

With a number of major hurdles to be overcome, personalized medicine, taking advantage of the great promising potential of 3D- and 2D-based culture system, is rapidly evolving. Recently, a first-in-human trial of intestinal organoids for the treatment of inflammatory bowel disease is about to be carried out [83]. More excitingly, the patient-derived organoids have been used to test and select effective drugs for cystic fibrosis [71,84]. The 2D-based monolayer culture also holds great promises in personalized medicine. There is no doubt that 3D organoid and 2D monolayer cultures provide complementary, efficient, and clinically relevant models for personalized medicine.

## Figures and Tables

**Table 1:** Characteristics of the described preclinical cancer models.

		3D-Based Culture System			2D-Based Culture System				
		Spheroids	PDX	Organoids	Monolayer				Air-Liquid Interface Culture
**Culture Technique**		Low-melting Agrose	Transplanted into immune-deficient mouse	Embended in Matrigel	Cell lines	Plate coated with Collagen I	Transwell coated with Collagen I	Plate coated with Matrigel	Seeded onto 3T3-J2 cells which cultured on transwell coated with 20% Matrigel
**Origin**		Cell lines	Patient-derived tumors	mIEC; hIEC; Patient-derived tumors	Cell lines	mIEC; hIEC	mIEC; hIEC	mIEC; hIEC; Tumors	hIEC; Tumors
**Key Features**	**In vivo-like complexity**	✗	✓	✓	✗	Lack of mature secretory lineage	Lack of mature secretory lineage	✓	✓
	**Stemness and multipotency**	-	✓	✓	-	✓	✓	✓	✓
	**Easily passaged**	✓	✓	✓	✓	✗	✗	✗	✓
**Applications**	**Cancer subtype modeling (gene manipulation)**	✓	✓	✓	✓	✓	✓	✓	✓
	**Host-pathogen interaction**	✗	✓	✗	✓	✓	✓	✓	✓
	**Co-culture**	✓	✓	✓	✓	✓	✓	✓	✓
	**High-throughput screening**	✓	✓	✓	✓	✓	✓	✓	✗
	**High-throughput imaging**	✓	✗	✗	✓	✓	✓	✓	✗
	**Time consumption for modeling**	Medium	Long	Medium	Short	Medium	Medium	Medium	Medium
	**Cost benefits**	Low	High	High	Low	Medium	Medium	Medium	High
**Reference**		[34]	[34]	[34,35,36,39,47,58,59,63]	[31,32,34]	[47,55,59]	[47,54,59]	[47,50,53,59]	[57]
